# Methotrexate reduces circulating Th17 cells and impairs plasmablast and memory B cell expansions following pneumococcal conjugate immunization in RA patients

**DOI:** 10.1038/s41598-021-88491-2

**Published:** 2021-04-28

**Authors:** Per Nived, Åsa Pettersson, Göran Jönsson, Anders A. Bengtsson, Bo Settergren, Lillemor Skattum, Åsa Johansson, Meliha C. Kapetanovic

**Affiliations:** 1 Department of Infectious Diseases, Central Hospital Kristianstad, J A Hedlunds väg 5, 291 85 Kristianstad, Sweden; 2 Department of Clinical Sciences Lund, Section of Rheumatology, Lund University, and Skåne University Hospital, Lund, Sweden; 3 Department of Clinical Sciences Lund, Nephrology, Lund University, Skåne University Hospital, Lund, Sweden; 4 Department of Clinical Sciences Lund, Section of Infectious Diseases, Lund University, and Skåne University Hospital, Lund, Sweden; 5 Department of Laboratory Medicine, Section of Microbiology, Immunology and Glycobiology, Lund University, and Clinical Immunology and Transfusion Medicine, Region Skåne, Lund, Sweden; 6 Department of Laboratory Medicine Lund, Hematology and Transfusion Medicine, Clinical Pathology, Lund University, Lund, Sweden

**Keywords:** Lymphocytes, Vaccines, Rheumatoid arthritis

## Abstract

Methotrexate (MTX) impairs antibody response after pneumococcal vaccination. We aimed to investigate differences in phenotypes of circulating B and T cells after pneumococcal conjugate vaccine (PCV) in rheumatoid arthritis (RA) patients on MTX (MTX group), RA without disease-modifying drugs (0DMARD), and controls (HC). MTX group (n = 11), 0DMARD (n = 12) and HC (n = 13) were studied. Blood samples were collected: before MTX, ≥ 4 weeks on stable MTX dose (prevaccination), and 7 days postvaccination (MTX group), and pre- and 7 days postvaccination (0DMARD and HC). Phenotypes of B- and T cell subsets were determined using flow cytometry. Serotype-specific IgG were quantified using multiplex bead assay, pre- and 4–6 weeks postvaccination. Concentrations of plasmablasts and switched memory B cells increased after PCV in HC (both *p* = 0.03) and the 0DMARD group (*p* = 0.01 and *p* = 0.02), but not in the MTX group. Postimmunization plasmablasts were lower in MTX group, compared to the 0DMARD group and HC (*p* = 0.002 and p < 0.001). Th17 cells decreased after MTX start (*p* = 0.02), and increased in HC after immunization (*p* = 0.01). Postimmunization plasmablasts correlated with mean antibody response ratio in all RA patients (R = 0.57, *p* = 0.035). Methotrexate reduced Th17 cells and blocked activation of plasmablasts and switched memory B cells following polysaccharide-protein conjugate antigen challenge in RA.

## Introduction

Rheumatoid arthritis (RA) is associated with an increased risk of infection^1^, and immunizations against vaccine-preventable diseases such as pneumococcal infection is important^2^. While immunosuppressive treatment with methotrexate (MTX) is well known to reduce antibody response after pneumococcal vaccination in RA^3,4^, the underlying mechanisms remain elusive.

The chronic joint inflammation characteristic of RA is often attributed to CD4^+^ T-helper (Th) cell mediated autoimmune pathology. In RA, Th1 cells are present in the synovium^5^, and stimulate macrophages to produce the pro-inflammatory cytokines including tumor necrosis factor (TNF)^6^. T-helper 17 (Th17) cells with the signature cytokine interleukin-17 (IL-17) can induce neutrophil inflammation in the synovial tissue and bone resorption in RA patients^6^. TNF-blockade and inhibition of T cell co-stimulation are successful treatments in RA^7,8^, but clinical trials have not shown efficacy of treatments targeting IL-17^9^.

B cells have an apparent role in the production of RA associated autoantibodies, such as rheumatoid factor (RF) and anti citrullinated peptide antibodies (ACPA), and B cell depletion therapy using anti-CD20 is efficacious for RA patients including those refractory to TNF-blockade^10^.

Pneumococcal conjugate vaccine (PCV) consisting of pneumococcal capsular polysaccharides conjugated to a carrier protein induces a T cell-dependent immune response^11^. Follicular T helper (Tfh) cells express chemokine receptor 5 (CXCR5), and specializes in providing help to B cells in germinal center reactions in secondary lymphoid organs, resulting in high-affinity antibody secreting plasma cells and memory B cells^12,13^. Peripheral blood CD45RA^-^ CXCR5^+^ Th cells are thought to represent a circulating memory compartment of Tfh cells (cmTfh)^14^. Recently activated cmTfh express surface markers inducible T cell co-stimulator (ICOS) and programmed cell death protein 1 (PD-1), and are most likely derived from germinal center reactions^15,16^.

Our group and several others have reported impaired antibody response to pneumococcal and influenza vaccines in RA patients treated with MTX^3,4,17–19^. However, the number of antigen specific plasmablasts in peripheral blood was not decreased^20^. Using vaccination with PCV as a model for antigen challenge, we aimed to investigate the underlying mechanisms by which MTX exerts its effect on the antibody response. We hypothesized that MTX treatment exerts a negative effect on the formation of germinal center (GC) Tfh cells after protein antigen stimulation, resulting in lower numbers of cmTfh cells after immunization with PCV. Thus, we conducted a study to investigate phenotypes of peripheral blood B and T cells, including memory Tfh cell subsets, before and after PCV in RA patients on stabile MTX dose compared to RA without DMARD, and healthy controls and thereby explore the mode of action of MTX in RA.

## Methods

### Patient inclusion

Adult RA patients either planned to start MTX treatment (MTX group), or without ongoing/planned DMARD treatment (0DMARD-group), at the Department of Rheumatology, Skåne University Hospital Lund were eligible for this study. Patients had to fulfil the American College of Rheumatology / European League Against Rheumatism criteria for RA^21^. At the time of inclusion, a rheumatologist performed a clinical examination and data were collected on disease and treatment characteristics and previous vaccinations using a structured protocol. Patients were excluded from the study if they had been treated with DMARD within 6 months, were treated with prednisolone > 15 mg/day, if they had previously received pneumococcal vaccine, had a history of allergic reaction at previous vaccinations, were pregnant, or had an ongoing infection. Healthy controls (HC) were recruited from the staff and relatives at the department of Rheumatology in Lund.

### Ethics approval and informed consent

The study was approved by the Regional Ethical Review Board at Lund University, Sweden (Dnr 2016/143). Consecutive patients fulfilling inclusion criteria were invited to participate in the study. Oral and written information was provided to all subjects who were invited to participate, and written informed consent was obtained from each participant before enrolment. All experiments were performed in accordance with relevant guidelines and regulations.

### Vaccination protocol

All participants received a single 0.5 mL dose of 13-valent pneumococcal conjugate vaccine (PCV13, Prevenar 13, Pfizer) administered as an intramuscular injection in the deltoid muscle. Patients in the 0DMARD group and HC received immunization at time of inclusion. Patients in the MTX group were immunized 6–12 weeks after start of methotrexate treatment and being on stable MTX dose for at least 4 weeks. At time of vaccination, a clinical examination was performed, and data was collected on disease activity.

### Flow cytometry and phenotypic characterization of B and T cells

In the MTX group, blood was drawn at inclusion (before MTX treatment), at vaccination (after 6–12 weeks on MTX), and 6–7 days after vaccination. In the 0DMARD and control groups, blood was drawn at vaccination (inclusion) and 6–7 days after.

Venous blood was obtained in heparin tubes, 4–6 mL (Becton Dickinson, BD, Vacutainer ref 369,622). The tubes were stored dark at room temperature and all samples were analyzed within 24 h. Red blood cells were lysed using 0.84% ammonium chloride for 10 min in room temperature, and washed once with PBS (without Mg^2+^ and Ca^2+^). The cell pellet was resuspended in 0.5% BSA/PBS and fluorochrome conjugated antibody mixes were added (see Table S1). The cells were incubated for 20 min, dark in room temperature and washed with PBS. The analyses were performed on FACS Aria Fusion (BD Bioscience) using FACS Diva software. Cell populations were identified according to the gating strategies described by Maecker et al.^22^. Definitions of B and T lymphocyte subsets are shown in Table S2, and gating strategies are illustrated in Figs. S1–S3.

### Serotype-specific antibodies

For all participants, serum samples were collected immediately before administration of PCV13 and 4–6 weeks after. Sera were frozen at −80 °C and later analyzed at the Department of Clinical Immunology, Lund, Sweden. Pneumococcal serotype-specific IgG concentrations were determined for 11 capsular serotypes (1, 3, 4, 5, 6B, 7F, 9V, 18C, 19A, 19F and 23F) included in PCV13, using an in-house multiplex fluorescent microsphere immunoassay (MFMI, Luminex) based on the procedure previously described by Lal et al.^23^. This method permits simultaneous measurement of antibodies to 11 serotypes in a single sample.

### Statistical analysis

Baseline results of the MTX and 0DMARD groups were treated as a single RA group because patients were not treated with any DMARDs at time of inclusion. Differences between two groups were evaluated using Mann–Whitney U-test. Paired data were analyzed using Wilcoxon matched-pairs signed rank test. Possible monotonic associations between two variables were examined using Spearman’s rank correlation. Positive antibody response was defined as at least twofold increase in pneumococcal serotype-specific IgG concentration in at least 50% of serotypes (at least 6 of 11 serotypes) after vaccination. The mean fold change was calculated to obtain a general measure of the antibody response for group comparisons and correlation purposes. Statistical calculations were performed with R 3.5.3 software.

## Results

Twelve RA patients without ongoing and without planned DMARD treatment (0DMARD group), 11 RA patient planned to start treatment with MTX, and 13 healthy controls were enrolled in this study. Disease activity at inclusion was moderate in the majority of 0DMARD patients (DAS28 median 4.7, range 2.9–7.0), and high in the MTX group (DAS28 median 5.7, range 4.6–7.5). Demographics, laboratory characteristics, clinical characteristics and ongoing treatments are summarized in Table 1. Most patients in the MTX group had new onset RA, with median disease duration 0.8 years (range 0–29), compared to median 5.0 years (range 0–54) in the 0DMARD group (*p* = 0.06).

**Table 1:** Demographic, clinical and laboratory characteristics and treatment.

	Healthy controls	RA 0DMARD	RA MTX
N	13	12	11
Age years, median (range)	40.0 (32.1–62.7)	56.6 (29.7–74.3)	63.1 (39.5–82.1)^1^
Gender, female	67%	67%	90%
RF positive	–	75%	100%
ACPA positive	–	92%	45%^2^
DAS28 at inclusion, median (range)	-	4.7 (2.9–7.0)	5.7 (4.6–7.5)^3^
DAS28 at vaccination, median (range)	–	4.7 (2.9–7.0)	4.6 (2.2–6.0)
CRP at vaccination, mg/L	0.7 (0.6–5.3)	3.9 (0.6–9.1)^4^	2.8 (0.6–14.0)^4^
ESR at vaccination, mm	5 (2–19)	25.5 (5–66)^5^	35 (4–64)^5^
Disease duration years, median (range)	–	5.4 (0.1–54)	0.8 (0.1–29)
Prednisolone dose, median (range) mg/day	0 (0)	0 (0–5)	0 (0–15)
MTX dose at vaccination, median (range) mg/week	0 (0)	0 (0)	20 (15–25)

^1^RA with MTX-start group were older than controls (*p* = 0.002).

^2^Percentage of ACPA positives were lower in RA with MTX-start compared to RA without DMARD (*p* = 0.02).

^3^DAS28 at inclusion was higher in group RA with MTX-start compared to RA without DMARD (*p* = 0.01).

^4^CRP at vaccination was higher in both RA groups compared to controls (p ≤ 0.001).

^5^ESR at vaccination was higher in both RA groups compared to controls (*p* < 0.01).

### Total B cells and B cell subsets at baseline

**Figure 1:**
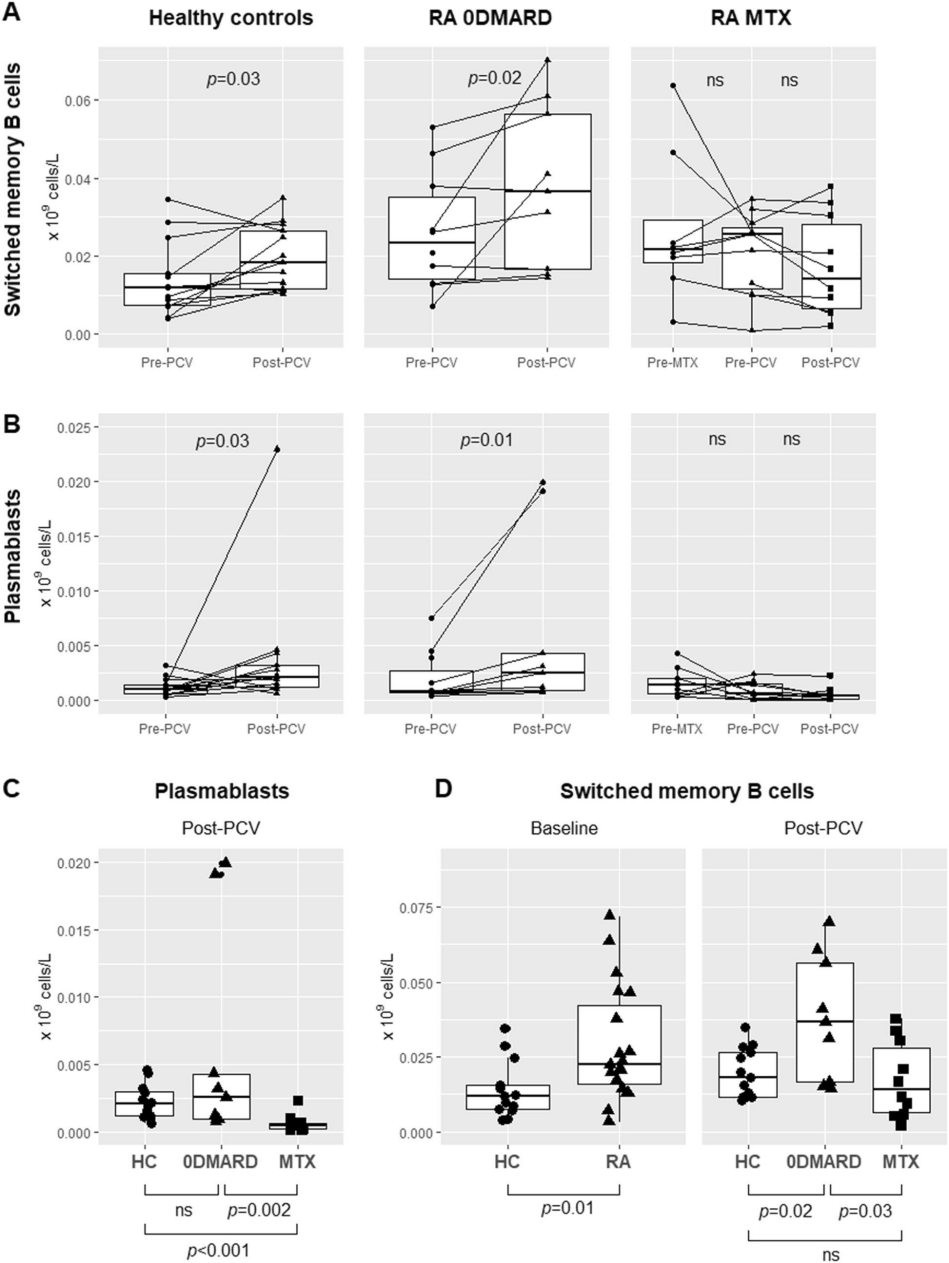
Concentrations of (**A**) switched memory B cells, and (**B**) plasmablasts in HC and RA 0DMARD groups: before and after PCV, and in RA MTX group: before start of MTX, before and after PCV. (**C**) Group comparison of plasmablast concentrations after PCV. (**D**) Group comparison of switched memory B cells at baseline and after PCV.

We determined different stages of peripheral blood B cell development, from naïve B cells migrating from the bone marrow to plasmablasts, i.e. the precursors of antibody-producing plasma cells. Subsets of B and T cells in RA groups and HC at baseline are shown in Table 2 (see definitions in Table S2). Switched memory B cells (% of B cells and concentrations) were higher in RA patients compared to HC (*p* = 0.034 and *p* = 0.010, Fig. 1D), but did not correlate with disease activity (DAS28). Total B cells (% of lymphocytes but not concentrations), were negatively correlated to disease duration (R = − 0.56, *p* = 0.01), but no correlation to age of RA patients was observed (R = − 0.38, *p* = 0.1). Double negative B cells are thought to represent a previously activated, non-functional, i.e. exhausted phenotype, which has been reported to increase (percentage but not numbers) in the elderly^24^. Double negative (exhausted) B cells (% of B cells) correlated with disease duration (R = 0.53, *p* = 0.017), and age of patients (R = 0.44, *p* = 0.05). Thus switched memory B cells might be more abundant in the blood of RA patients, and percentages of circulating total B cells seems to decrease in long-standing disease. A subset of cells with exhausted phenotype accumulate and might in part reflect normal aging (immune senescence).

**Table 2:** Total lymphocytes and B and T cells with subsets at baseline.

	Healthy controls	RA 0DMARD	RA MTX
Lymphocytes, × 10^9^ cells/L^1^	1.4 (1.0–2.4)	1.8 (0.9–2.5)	1.7 (0.7–2.5)
**B cells**, % of lymphocytes	5.7 (2.6–11.2)	4.8 (1.3–14.7)	10.2 (1.8–15.7)
Naïve, % of B cells	52.4 (40.9–69.2)	51.3 (18.9–74.8)	55.6 (24.7–82.5)
Transitional, % of naïve	7.9 (1.5–15.0)	6.5 (0.2–14.2)	3.3 (0.3–14.7)
Preswitch memory, % of B cells	7.0 (2.2–25.9)	10.0 (2.0–21.3)	6.0 (1.1–14.6)
Switched memory, % of B cells	14.8 (6.1–27.7)	**27.0 (9.4–57.4)**^**2**^	23.4 (4.5–56.6)
Plasmablasts, % of switched	9.5 (2.4–30.9)	6.2 (1.9–14.3)	6.8 (1.6–11.3)
Exhausted, % of B cells	22.5 (4.9–36.1)	11.5 (4.0–24.9)	12.2 (6.1–30.4)
**T cells**, % of lymphocytes	72.7 (54.2–86.8)	75.4 (55.7–87.4)	72.0 (44.4–85.5)
HLA-DR^+^, % of T cells	1.9 (0.4–7.5)	1.8 (0.4–3.5)	1.7 (0.6–6.5)
CD38^+^, % of T cells	9.3 (2.5–31.3)	14.9 (3.1–28.9)	8.5 (2.5–37.1)
NKT cells, % of T cells	7.5 (0.8–42.2)	12.2 (0.9–48.0)	9.4 (2.2–20.7)
CD4^+^ Th, % of T cells	66.4 (46.1–81.5)	51.6 (31.0–83.6)	66.6 (48.6–77.7)
Naïve Th, % of CD4^+^	49.0 (10.5–58.5)	38.9 (11.6–73.3)	42.5 (13.7–64.3)
TEMRA, % of CD4^+^	20.8 (8.4–62.0)	20.2 (11.9–67.3)	16.3 (4.2–43.0)
Central memory, % of CD4^+^	8.3 (0.1–22.1)	9.1 (3.4–21.8)	10.5 (4.5–47.8)
Effector memory, % of CD4^+^	18.4 (0.1–32.6)	20.2 (10.9–54.8)	19.7 (14.1–48.2)
Th1, % of CD4^+^	10.1 (5.0–23.5)	8.4 (4.0–15.6)	11.6 (2.2–19.5)
Th2, % of CD4^+^	18.7 (6.0–24.7)	16.8 (0.9–26.4)	15.2 (6.1–65.4)
Th17, % of CD4^+^	18.7 (8.6–28.7)	17.1 (8.7–33.7)	18.4 (7.4–56.0)
Treg, % of CD4^+^	2.8 (0.3–5.7)	1.2 (0.5–5.1)	2.0 (0.3–9.6)
Activated Treg, % of Treg cells	19.6 (7.4–32.3)	20.6 (8.4–43.0)	21.7 (1.9–52.2)
cmTfh, % of CD4^+^CD45RO^+^ T cells	21.3 (10.2–30.9)	22.7 (9.5–29.0)	14.3 (11.7–74.9)
cmTfh1, % of cmTfh cells	16.8 (9.8–24.5)	17.6 (11.3–23.6)	16.5 (9.7–29.4)
cmTfh2, % of cmTfh cells	17.5 (11.4–24.3)	18.8 (10.1–35.1)	22.9 (7.6–32.6)
cmTfh17, % of cmTfh cells	45.8 (35.8–60.5)	42.7 (27.0–57.5)	41.8 (26.4–58.5)
PD1^+^ICOS^+^ cmTfh, % of cmTfh cells	1.3 (0.3–5.1)	2.2 (0.1–7.5)	1.4 (0–3.8)
Tph, % of CD4^+^	0.7 (0.1–0.9)	0.8 (0.2–2.0)	0.9 (0.3–2.6)

^1^All results are presented as median (range).

^2^Switched memory B cells were higher in RA 0DMARD and all RA patients compared to HC (*p* < 0.01 and *p* = 0.02).

### Dynamics of switched memory B cells and plasmablasts after pneumococcal immunization in relation to RA and methotrexate treatment

We continued by investigating possible effects of initiation of MTX and immunization with PCV on the numbers of circulating switched memory B cells and plasmablasts, thought to arise from GC-reactions. Concentrations of circulating switched memory B cells increased after pneumococcal vaccination in HC and in the 0DMARD group (respectively, *p* = 0.03 and *p* = 0.02), but no changes were seen in the MTX group (Fig. 1A). After vaccination, switched memory B cells were lower in the MTX group compared to the 0DMARD group (*p* = 0.03, Fig. 1D), but not compared to HC (*p* = 0.5). Switched memory B cells were higher in the 0DMARD group compared to HC, after vaccination (*p* = 0.02). Similarily, plasmablast concentrations increased after immunization in HC and the 0DMARD group (*p* = 0.03 and *p* = 0.01, respectively), but not in the MTX group (Fig. 1B). After vaccination, plasmablasts were lower in the MTX group compared to the 0DMARD group (*p* = 0.002), and HC (*p* < 0.001, Fig. 1C). In summary, while switched memory B cells and plasmablasts expanded in HC and the 0DMARD group after immunization, no such effect was seen in MTX treated patients.

### Total T cells and subsets at baseline

Further, circulating T cells in RA and HC at baseline were characterized by expression of activation markers HLA-DR or CD38, CD4^+^ T helper cells by stages of differentiation, and functional subsets Th1, 2, 17, Treg, Tfh, and T peripheral helper (Tph) cells. Finally, cmTfh cells were subgrouped into cmTfh1, -2 or -17 subsets with differential expression of activation markers PD-1 and ICOS. Subsets of T cells in RA groups and HC at baseline are shown in Table 2 (see definitions in Table S2). No differences in T cell subsets were found at baseline.

### Activated CD38^+^ T cells and Th1, -2 and -17 cell subsets in relation to immunization with pneumococcal conjugate vaccine and methotrexate treatment

**Figure 2:**
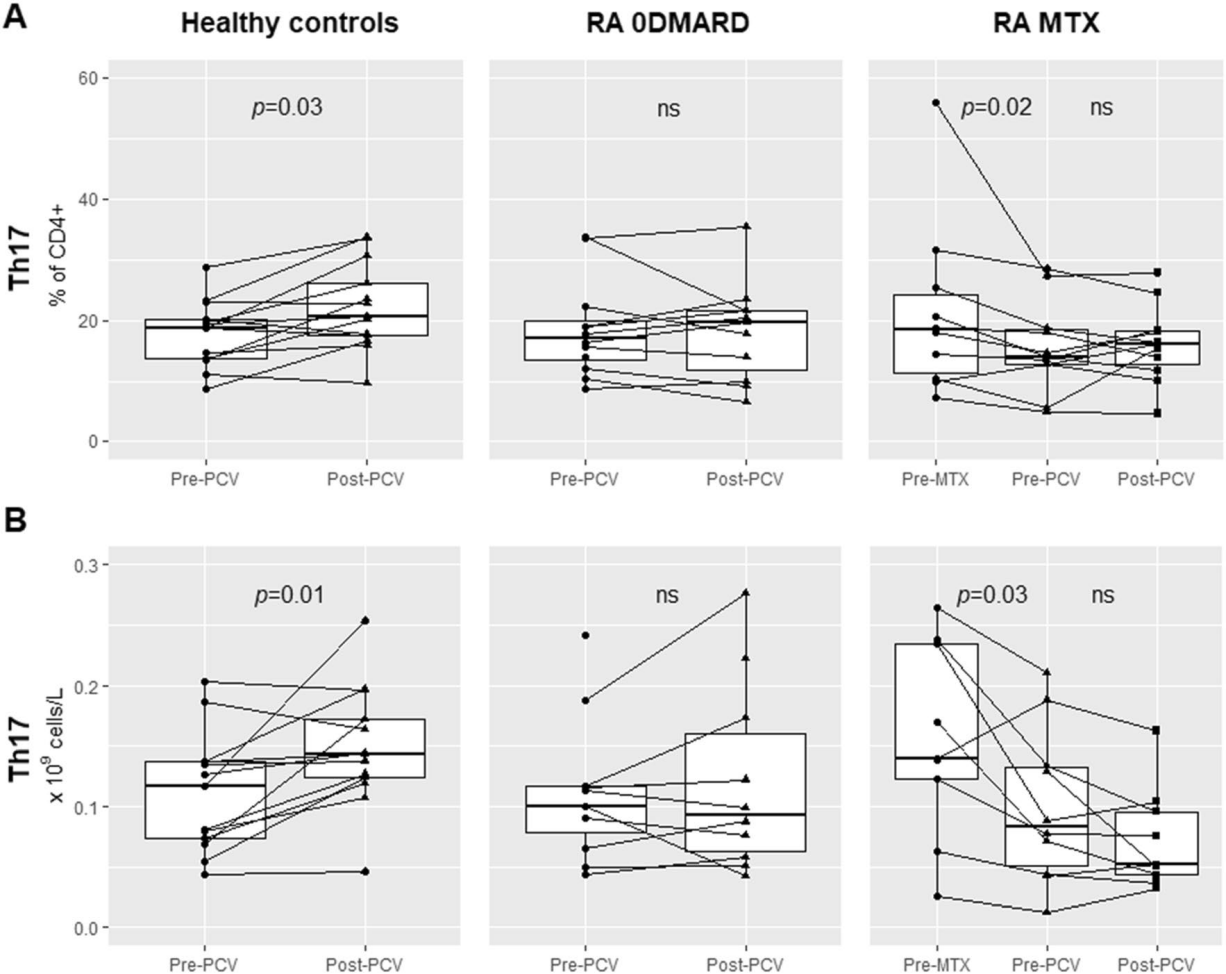
T helper 17 cells in % of CD4^+^ (**A**), and concentrations (**B**), in HC and RA 0DMARD groups: before and after PCV, and in MTX group: before start of MTX, before and after PCV.

The next step was to explore possible effects of MTX, and immunization on blood T cell activation and distribution of Th cell subsets. In the MTX group, initiation of methotrexate treatment resulted in decreased Th17 cell frequencies (% of CD4^+^) and concentrations (*p* = 0.02 and *p* = 0.03, Fig. 2). In HC, vaccination resulted in increased activated CD38 + T cell frequencies (% of T cells, *p* = 0.02) and concentrations (*p* = 0.006), and increased Th17 cell frequencies (% of CD4^+^, *p* = 0.03) and concentrations (*p* = 0.01, Fig. 2).

Before immunization, % and concentrations of Th17 cells were numerically but not statistically significant lower in the MTX group compared to HC and 0DMARD group. After vaccination, % and concentrations of Th17 did not increase significantly in the two RA groups. MTX group had lower postimmunization Th17 cell concentrations compared to HC (*p* = 0.02), but not compared to 0DMARD (*p* = 0.14). In addtion, % of Th17 cells was lowest in the MTX group but the difference between the groups did not reach statisticaly signicant level (Fig. 2). Thus both RA disease itself (i.e. the immunological disturbance as a part of the disease itself) and MTX may have a negativ impact on the peripheral blood Th17 cells, i.e. a T helper cell subset involved in the defense against extracellular bacteria, especially at mucosa^12^, and proposed to have a role in vaccine-induced memory responses^25^.

### Circulating T follicular helper cell subsets in relation to methotrexate and immunization

**Figure 3:**
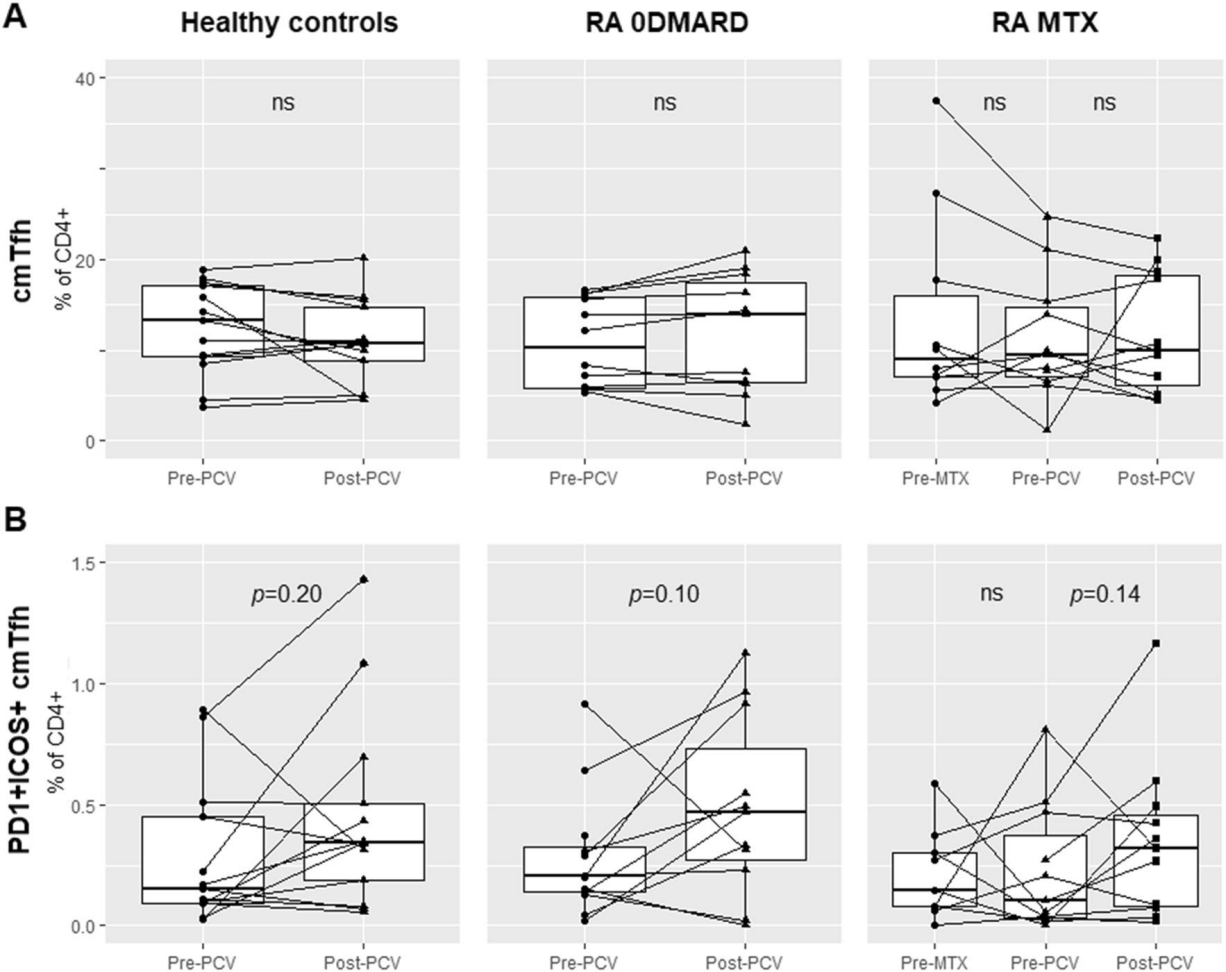
Circulating memory T follicular helper cells (cmTfh) in % of CD4^+^ (**A**), and activated, PD-1^+^ICOS^+^ cmTfh (**B**), in HC and RA 0DMARD groups: before and after PCV, and in MTX group: before start of MTX, before and after PCV.

Our initial hypothesis was that MTX might exert a negative effect on B cell helping Tfh cells in GC reactions, thus leading to reduced cmTfh cells after antigen stimulation with PCV in MTX treated RA patients. However, no effects of MTX on total or activated (ICOS^+^ PD-1^+^) cmTfh cells were observed (Fig. 3). Frequencies of total cmTfh cells (% of CD4^+^ cells) were unchanged after immunization in all groups (Fig. 3A). Although non significant, activated cmTfh cells (% of CD4^+^) increased after vaccination in 8 of 11 patients in the 0DMARD group (*p* = 0.10), 7 of 11 patients in the MTX group (*p* = 0.14), and in 7 of 12 HC (*p* = 0.20, Fig. 3B). Percentage of cmTfh1 cells decreased and cmTfh2 cells increased after vaccination in HC, but no changes were observed in the RA groups (Fig. S4A,B). In HC, increased cmTfh17 cells (% of Tfh cells) was seen after PCV (*p* = 0.03), but no significant changes were seen in 0DMARD (Fig. S4C). In the MTX group numbers of cmTfh17 cells decreased after vaccination (*p* = 0.03), but there was no difference in % of cmTfh cells (Fig. S4C). We did not find any effects of MTX treatment on cmTfh cell subsets after immunization, but activated cmTfh cells tended to increase in most participants following immunization.

### Pneumococcal antibody concentrations

As a final step, pneumococcal serotype-specific IgG concentrations of 11 PCV13-serotypes were analyzed, before and 4–6 weeks after immunization. Positive antibody responses (≥ twofold increase in ≥ 6 serotypes) were seen in 90% of HC participants, 87.5% of the 0DMARD group, and 56% of the MTX group. Number of serotypes with positive antibody responses were lower in MTX group compared to HC (*p* = 0.003), and 0DMARD (*p* = 0.04). Serotype-specific antibody response is shown in Fig. S5. Of all RA patients, responders had higher post PCV plasmablast concentrations compared to nonresponders (*p* = 0.04). Mean fold change in pneumococcal IgG concentrations correlated with plasmablast concentrations in all participants (R = 0.52, *p* = 0.011), and all RA patients (R = 0.57, *p* = 0.035). Although non-specific plasmablasts were measured in this study, these cells correlated to the pneumococcal antigen-specific antibody response.

## Discussion

We have demonstrated expansions of circulating plasmablasts and switched memory B cells seven days after immunization with pneumococcal conjugate vaccine, in HC and RA patients without DMARDs, but these were absent in MTX treated patients. Further, initiation of MTX treatment reduced Th17 cells in blood. We propose that the absent expansions of circulating plasmablasts and switched memory B cells after PCV during MTX treatment, is accompanied by impaired clonal expansion of pneumococcal antigen-specific B cells. Theoretically, the blocking action of MTX could be a direct inhibition of B cell activation, and possibly also indirect action via T helper cell capacity, or a combination of B and T cell effects. The main function of Th17 cells is in the defense against extracellular bacteria, e.g. pneumococci, and fungi on mucosal surfaces^12^, and the Th17 cell subset may be important for vaccine induced memory immune responses^25^. MTX induced inhibition of B cell responses is consistent with previous findings of impaired short-term antibody responses, and low persistence of antibodies 1.5 years, after immunization with T cell independent pneumococcal polysaccharide vaccine^3,26^. On the other hand, MTX is also known to impair antibody responses after T cell dependent PCV, and influenza virus vaccine in arthritis patients^4,19^. In contrast to the present findings, a previous report from our group found no reductions in total, or antigen-specific plasmablasts after PCV in MTX treated RA patients^20^. However, in our first study plasmablasts were enumerated using a different method (ELISPOT) that might be one of explanations for the diverging results.

In the present study we observed reductions of blood Th17 cells 6–12 weeks after start of MTX treatment. Szalay et al. reported similar results, i.e. decreased prevalence of Th17 cells, 4 weeks after starting MTX treatment in 19 treatment-naïve early RA patients^27^. In vitro addition of MTX to peripheral blood mononuclear cells from RA patients also resulted in decreased Th17 cells^28^. However, in spite of a significant reduction of circulating Th17 cells after induction of MTX, Th17 cells before vaccination were not significantly lower in the MTX group compared to HC or 0DMARD patients. These results indicate that Th17 cells do not play a crucial role in the impaired vaccine response observed among MTX treated patients with RA.

We also found possible disturbances in T cell responses of untreated RA patients, as illustrated by absent expansions of circulating CD38^+^ activated T cells, and Th17 cells after PCV immunization in RA patients compared to healthy controls. Shen et al. previously demonstrated increased frequencies of IL-17 producing T cells in PBMCs from RA patients and strong correlation with disease activity^29^, but in contrast to our study they used ex vivo mitogen stimulation.

Switched memory B cells in blood were increased in untreated RA, compared to HC, suggesting activation of these cells in RA, although no association with disease activity was seen. T follicular helper cells provides critical help to B cells in GC reactions during the T cell dependent immune response to pneumococcal conjugate vaccine. In the circulating memory compartment of Tfh cells, subsets cmTfh17 and cmTfh2, but not cmTfh1, are considered efficient B cell helpers^14^. Circulating memory Tfh17 cells promote IgG and IgA, and cmTfh2 cells IgG and IgE class switch in B cells. Circulating memory PD-1^+^ICOS^+^ Tfh cells are most likely recently activated Tfh cells derived from GC reactions^15,16^, and although non-significant, frequencies of these cells tended to increase in the majority of patients and controls.

In the oncological setting, high doses of the folate antagonist MTX cause disruption of purine synthesis, and subsequent apoptosis of malignant cells. Although MTX has been the predominant treatment for rheumatoid arthritis patients since three decades, the mechanisms behind its low dose (15–25 mg/week) anti-inflammatory effects are still not fully understood^30^. Proposed effects of MTX on T cells include increased susceptibility to apoptosis, inhibition of the transcription factor nuclear factor kappa B (NF-κB), and promotion of immunosuppressive adenosine secretion from Tregs, causing inhibition of T cell activity^31^. Bitoun et al. have reported a similar effect on B cells, where interaction between MTX and B cell activation factor (BAFF) promotes adenosine release from regulatory B cells, which reduce immunization against therapeutic monoclonal antibodies^32^. Park et al. demonstrated a negative impact on antibody responses to seasonal influenza vaccine in MTX treated patients with high BAFF levels, compared to patients with low BAFF-levels^33^. Our findings on the absent expansions of circulating plasmablasts and switched memory B cells, and consequently lower antibody response after immunization with PCV in MTX treated patients are in line with the above mentioned studies^32,33^. Although positive antibody responses to at least 6 serotypes were seen in 56% of MTX treated RA patients, these patients responded to significantly fewer pneumococcal serotypes compared to HC and untreated RA patients. Taken together, these results suggest that effect on plasmablasts and memory B cells in combination with no increase in activated CD38^+^ T cells and decrease of Th17 cells could be important parts of the mode of action of MTX in RA.

This study had some limitations. Due to small sample size and nonrandomized design, results should be interpreted with some caution. Definitions of T helper cell subset were based on current knowledge when the study was designed, but could be improved in future studies. Furthermore, we did not enumerate pneumococcal antigen-specific B or T cells, but on the other hand we measured distinct effects on nonspecific plasmablasts after vaccination that showed a moderately strong correlation to the specific pneumococcal antibody response. Future studies should be designed to measure the effect of MTX on pneumococcal specific immune cells. Further, MTX RA group were significantly older compared with HC group, which possibly contributed to both lower lymphocyte responses and antibody responses in this group.

## Conclusions

Methotrexate blocked expansions of circulating switched memory B cells and plasmablasts after antigen stimulation with pneumococcal conjugate vaccine in patients with rheumatoid arthritis. Initiation of MTX treatment reduced Th17 lineage cells, an effect that may have implications for T-cell mediated immune responses after vaccination. Further studies are needed to elucidate the mechanisms behind impaired T cell dependent vaccine responses in MTX treated RA patients.

## Supplementary Information

Supplementary Information.
