# Supplementary material for: Methotrexate reduces circulating Th17 cells and impairs plasmablast and memory B cell expansions following pneumococcal conjugate immunization in RA patients
Source: Sci Rep. 2021 Apr 28;11:9199. doi: 10.1038/s41598-021-88491-2 (PMC8080705; doi:10.1038/s41598-021-88491-2)

**Methotrexate reduces circulating Th17 cells and impairs plasmablast and memory B cell expansions following pneumococcal conjugate immunization in RA patients**

Per Nived^1,2^, Åsa Pettersson^3^, Göran Jönsson^4^, Anders A Bengtsson^2^, Bo Settergren^1^, Lillemor Skattum^5^, Åsa Johansson^6^, Meliha C Kapetanovic^2^

^1^Department of Infectious Diseases, Central Hospital, Kristianstad, Sweden.

^2^Department of Clinical Sciences Lund, Section of Rheumatology, Lund University, and Skåne University Hospital, Lund, Sweden.

^3^Department of Clinical Sciences Lund, Nephrology, Lund University, Skåne University Hospital, Lund, Sweden.

^4^Department of Clinical Sciences Lund, Section of Infectious Diseases, Lund University, and Skåne University Hospital, Lund, Sweden.

^5^Department of Laboratory Medicine, Section of Microbiology, Immunology and Glycobiology, Lund University, and Clinical Immunology and Transfusion Medicine, Region Skåne, Lund, Sweden.

^6^Department of Laboratory Medicine Lund, Hematology and Transfusion Medicine, Lund University, Clinical pathology, Lund, Sweden.

**Corresponding author:**

Per Nived, MD

Department of Infectious Diseases, Central Hospital Kristianstad

J A Hedlunds väg 5

SE-291 85 Kristianstad, Sweden

Email: per.nived@med.lu.se

**Supplementary Table S1.** Antibody mix.

**T cells**

*Antibody Flourochrome Supplier/art no Clone*

CD25 PerCP Cy5.5 BD 560503 M-A251

CD183 Alexa Flour 488 Bio Legend 353710 GO25H7

CD196 PE Bio Legend 353410 GO34E3

HLA-DR APC H7/Cy7 BD 561358 G46-6

CD3 Alexa 700 BD 557943 UCHT1

CD197 Alexa Flour 647 BD 557734 3D12

CD194 BV785/786 BD 744141 1G1

CD45RO BV650 BD 563750 UCHL1

CD8 V500 BD 560774 RPA-T8

CD4 V450 BD 560345 RPA-T4

CD45RA PE Cy7 BD 560675 HI100

CD127 CF594 BD 562397 HIL-7R-M21

**B cells**

*Antibody Flourochrome Supplier/art no Clone*

CD19 PerCPCy5.5 BD 561295 HIB19

CD24 FITC BD 560992 ML5

HLA-DR PE BD 555812 G46-6

CD27 APC H7 BD 560222 M-T271

CD3 Alexa Flour 700 BD 557943 UCHT1

CD56 Alexa Flour 647 BD557711 B159

CD45 V500 BD560779 H130

IgD V450 BD 561309 IA6-2

CD38 PE Cy7 BD335825 HB7

**cTfh cells**

*Antibody Flourochrome Supplier/art no Clone*

CD185 PerCPCy5.5 BD 560831 RF8B2

CD183 Alexa Flour 488 BioLegend 353710 GO25H7

CD196 PE BioLegend 353410 G034E3

HLA-DR APC-H7 BD 561358 G46-6

CD3 Alexa Flour 700 BD 557943 UCHT1

CD197 Alexa Flour 647 BD 557734 3D12

CD278 BV786 BD 741017 DX29

CD45RO BV650 BD 563750 UCHL1

CD8 V500 BD 560774 RPA-T8

CD4 V450 BD 560345 RPA-T4

CD45RA PECy7 BD 560675 HI100

CD279 CF594 BD 565204 EH12.1 **Supplementary Table S2.** Definitions of B- and T-lymphocyte subsets in peripheral blood.

Phenotype Cell surface markers

**B cell CD19+**

Naive CD19+ IgD+ CD27-

Transitional CD19+ IgD+ CD27- CD24+ CD38+

Pre-switch memory CD19+ IgD+ CD27+

Switched memory CD19+ IgD- CD27+

Plasmablasts CD19+ IgD- CD27+ CD38++ CD24-

Exhausted (double negative) CD19+ IgD- CD27-

**T cell CD3+**

Activated CD38+ CD3+ CD38+

Activated HLA-DR+ CD3+ HLA-DR+

NKT cell CD3+ CD56+

T helper (Th) cell CD3+ CD4+

Naive CD3+ CD4+ CD45RA+ CCR7+

Central memory (Tcm) CD3+ CD4+ CD45RA- CCR7+

Effector memory (Tem) CD3+ CD4+ CD45RA- CCR7-

Effector memory re-expressing

CD45RA (TEMRA) CD3+ CD4+ CD45RA+ CCR7-

Th1 CD3+ CD4+ CD45RO+ CD183+ CD196-

Th2   CD3+ CD4+ CD45RO+ CD183- CD196-

Th17 CD3+ CD4+ CD45RO+ CD183- CD196+

Treg CD3+ CD4+ CD45RO+ CCR4+ CD25^high^ CD127-

Activated Treg CD3+ CD4+ CD45RO+ CCR4+ CD25^high^ CD127- HLA-DR+

cmTfh CD3+ CD4+ CD45RO+ CXCR5+

cmTfh1 CD3+ CD4+ CD45RO+ CXCR5+ CD183+ CD196-

cmTfh2 CD3+ CD4+ CD45RO+ CXCR5+ CD183- CD196-

cmTfh17 CD3+ CD4+ CD45RO+ CXCR5+ CD183- CD196+

Activated cmTfh CD3+ CD4+ CD45RO+ CXCR5+ PD1+ ICOS+

Tph CD3+ CD4+ CD45RO+ CXCR5- PD1^high^

**Supplementary Figure S1.** Gating strategies for circulating memory T follicular helper cells (cmTfh) and T peripheral helper cells (Tph).


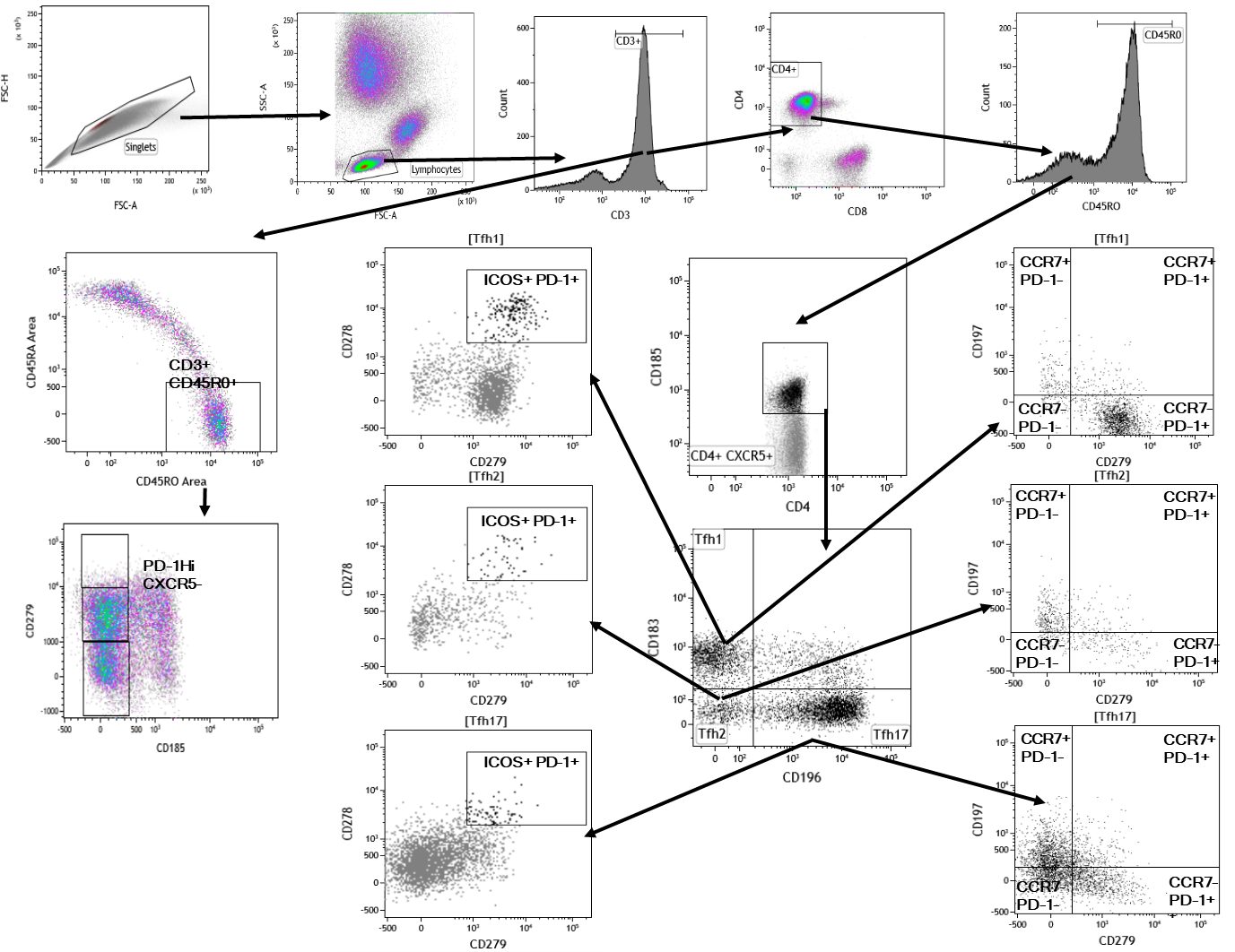


**Supplementary Figure S2.** Gating strategies for B cells, NK cells and activated T cells. The gates for CD38+ and HLA-DR+ T cells were based on the location of HLA-DR+ and CD38+ B cells.


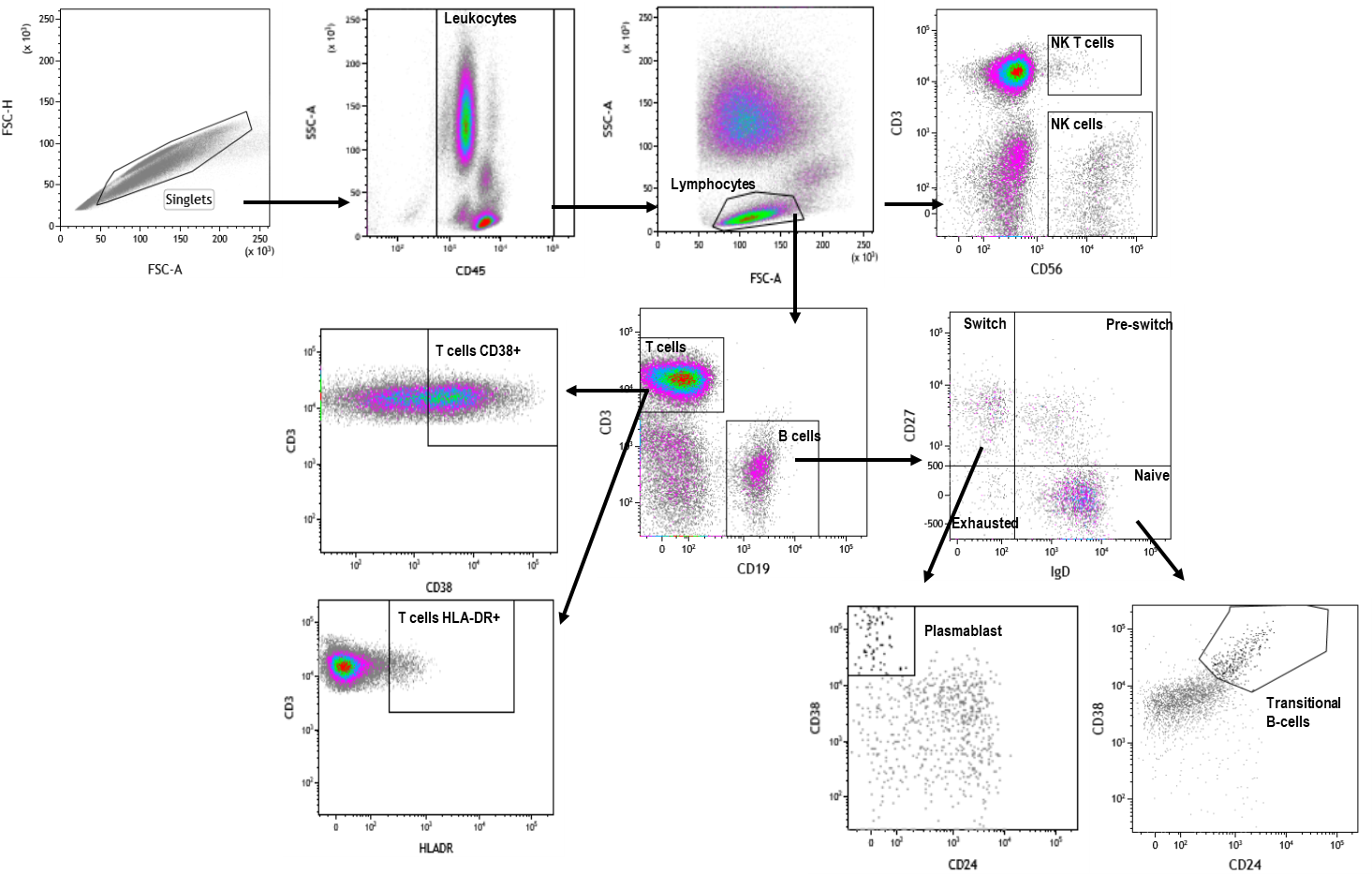


**Supplementary Figure S3.** Gating strategies for CD4^+^ T helper (Th) cell stages of differentiation, and subsets Th1, Th2, Th17, and T regulatory cells (Treg).

**
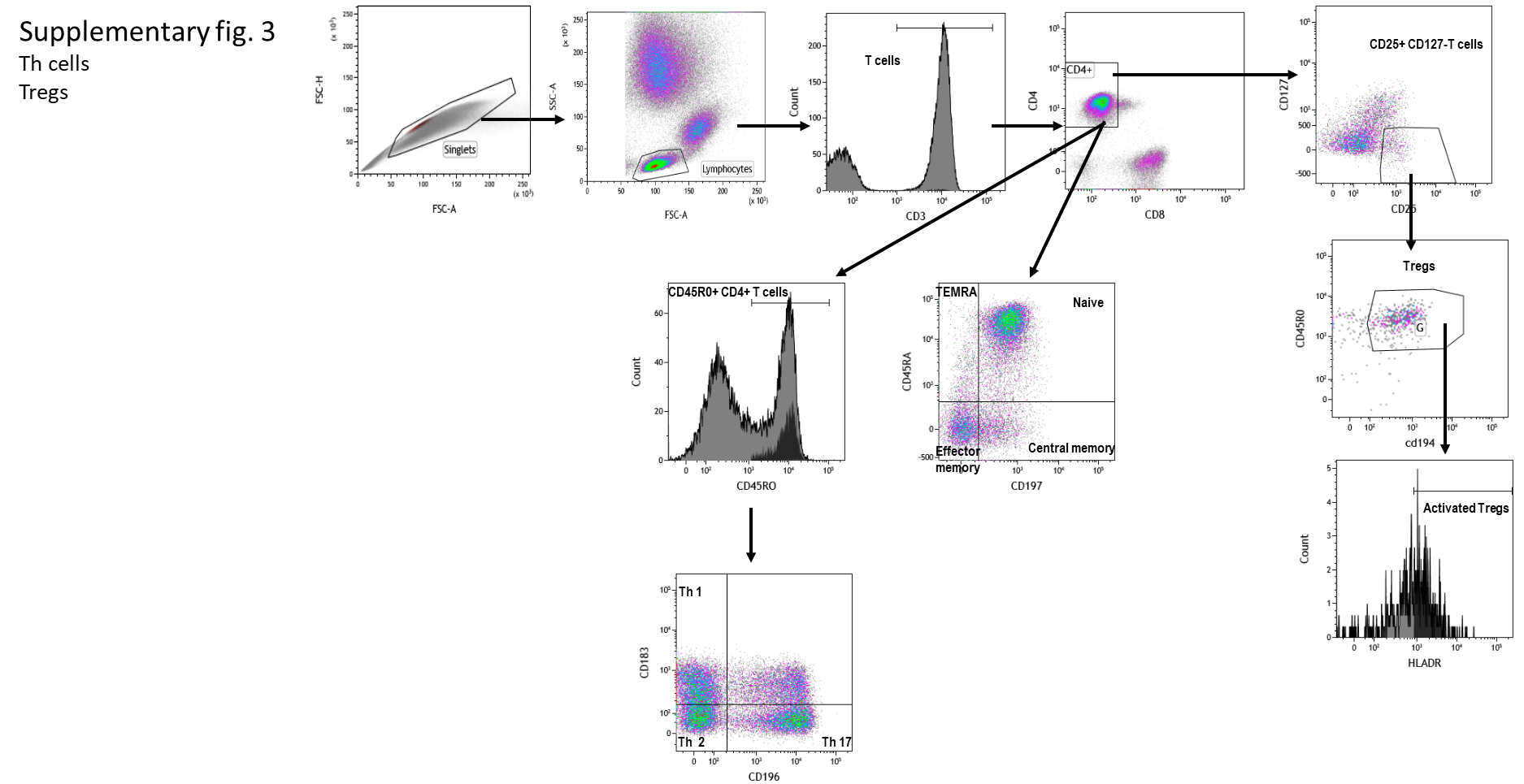
**

**Supplementary Figure S4.** Percentages of circulating memory Tfh1 (A), Tfh2 (B), and Tfh17 (C) subsets, in HC and RA 0DMARD groups: before and after PCV, and in MTX group: before start of MTX, before and after PCV.


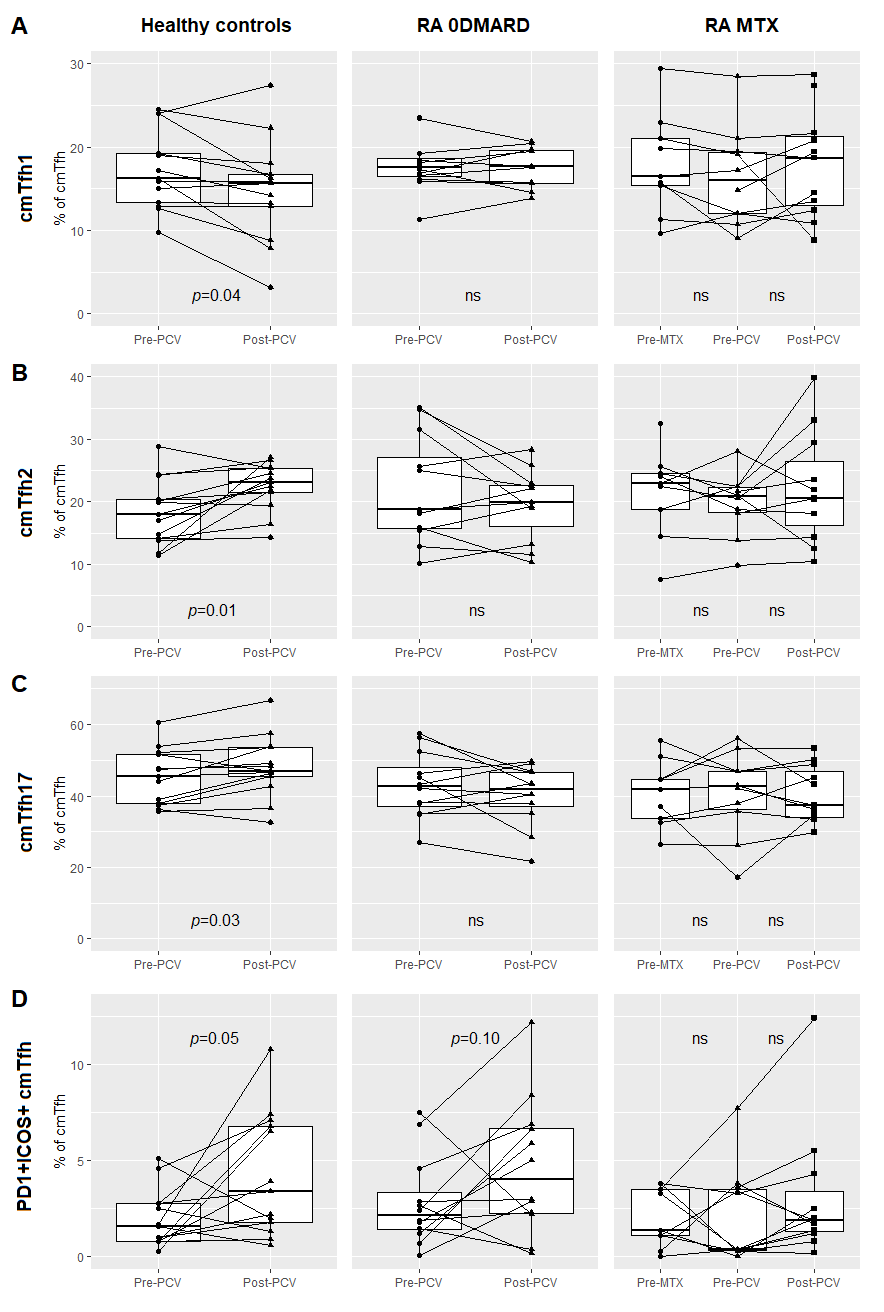


**Supplementary Figure S5.** Serotype-specific pneumococcal antibody response (% with antibody response ratio ≥2) in healthy controls, RA 0DMARD, and RA MTX groups, after immunization with PCV13.


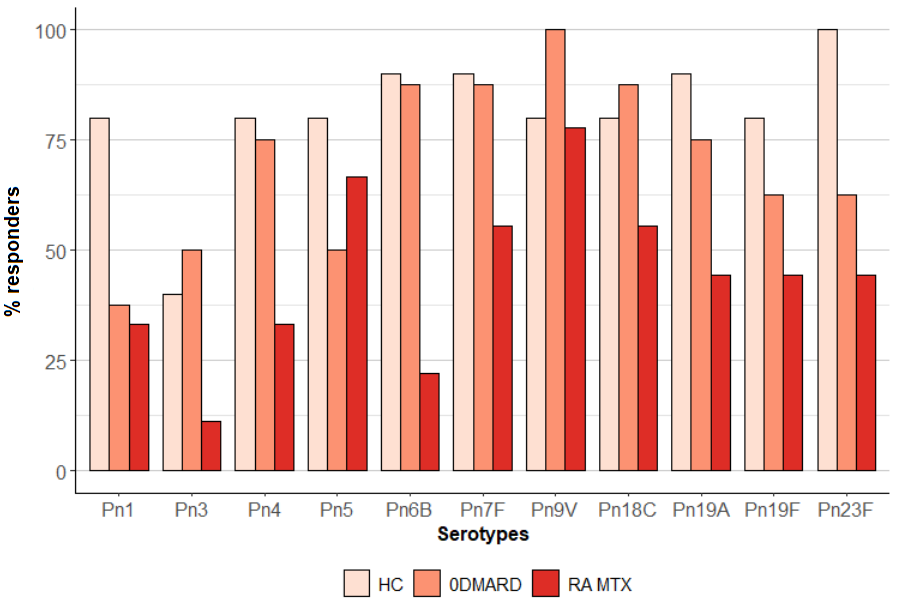

Supplement: Supplementary file 1 — Supplementary Information. [file 41598_2021_88491_MOESM1_ESM.docx]
